# Diagnostic accuracy of different computer-aided diagnostic systems for prostate cancer based on magnetic resonance imaging: A systematic review with diagnostic meta-analysis

**DOI:** 10.1097/MD.0000000000023817

**Published:** 2021-01-22

**Authors:** Xiping Xing, Xinke Zhao, Huiping Wei, Yingdong Li

**Affiliations:** a Affiliated hospital of Gansu University of Chinese Medicine; b Gansu University of Traditional Chinese Medicine, Lanzhou, China.

**Keywords:** Computer-aided detection, diagnostic accuracy, magnetic resonance imaging, meta-analysis, prostate cancer, systematic review

## Abstract

**Background:**

Computer-aided detection (CAD) system for accurate and automated prostate cancer (PCa) diagnosis have been developed, however, the diagnostic test accuracy of different CAD systems is still controversial. This systematic review aimed to assess the diagnostic accuracy of CAD systems based on magnetic resonance imaging for PCa.

**Methods:**

Cochrane library, PubMed, EMBASE and China Biology Medicine disc were systematically searched until March 2019 for original diagnostic studies. Two independent reviewers selected studies on CAD based on magnetic resonance imaging diagnosis of PCa and extracted the requisite data. Pooled sensitivity, specificity, and the area under the summary receiver operating characteristic curve were calculated to estimate the diagnostic accuracy of CAD system.

**Results:**

Fifteen studies involving 1945 patients were included in our analysis. The diagnostic meta-analysis showed that overall sensitivity of CAD system ranged from 0.47 to 1.00 and, specificity from 0.47 to 0.89. The pooled sensitivity of CAD system was 0.87 (95% CI: 0.76–0.94), pooled specificity 0.76 (95% CI: 0.62–0.85), and the area under curve (AUC) 0.89 (95% CI: 0.86–0.91). Subgroup analysis showed that the support vector machines produced the best AUC among the CAD classifiers, with sensitivity ranging from 0.87 to 0.92, and specificity from 0.47 to 0.95. Among different zones of prostate, CAD system produced the best AUC in the transitional zone than the peripheral zone and central gland; sensitivity ranged from 0.89 to 1.00, and specificity from 0.38 to 0.85.

**Conclusions:**

CAD system can help improve the diagnostic accuracy of PCa especially using the support vector machines classifier. Whether the performance of the CAD system depends on the specific locations of the prostate needs further investigation.

## 1 Introduction

Prostate cancer (PCa) is the most frequently diagnosed cancer among men in over one-half (105 of 185) of the countries of the world, notably in the Americas, Northern and Western Europe. And it is the leading cause of cancer death among men in 46 countries, particularly in Sub-Saharan Africa and the Caribbean.^[1–3]^ It is estimated that almost1.3 million new cases of PCa and 359,000 associated deaths worldwide in 2018, accounting for 7.1% of the total new cancers diagnosed worldwide, ranking as the second most frequent cancer and the fifth leading cause of cancer death in men.^[1,3]^ Accurate and early detection of PCa can ensure that patients receive treatment immediately, which can help prevent them from further progression and metastasis, and improve their survival rate.^[4]^

Therefore, reliable and early detection of PCa has become an important priority in the field of urologic oncology. For the past 25 years, Prostate-specific antigen has always been the gold standard for the diagnosis of PCa, followed by transrectal ultrasound -guided biopsy, which has decreased PCa related mortality by 20% to 30%,^[5]^ this method however with substantial diagnostic errors in undersampling and understaging PCa, resulting in overtreatment related morbidity such as incontinence and impotence.^[6,7]^ Over the past decade, multi-parametric magnetic resonance imaging (mp-MRI), has become the dominant non-invasive diagnostic tool for diagnosing and grading PCa.^[8]^ 3 Tesla mp-MRI enables detection of 50% of all PCa lesions and 80% of clinically significant lesions.^[9]^

However, 1 of the main limitations of the mp-MRI is that its interpretation requires experienced radiologists capable of analysing data extracted from the different MR sequences. The interpretation may lead to high inter- and intra-reader variability in diagnosis.^[10]^ Automated and accurate PCa detection from mp-MRI sequences could minimize the time required for interpreting the images, alleviating the requirement for expertise in radiological reading, and thus reducing the risk of over- and under-treatment and enabling large-scale PCa screening.

In the past decade, several computer-aided systems^[11–14]^ (CADs) for accurate and automated PCa detection and diagnosis have been developed. An increasing number of studies indicated that the CAD systems have the potential to support the radiologist by indicating suspicious regions and reducing oversight and perception errors^[15]^. In addition, some CAD applications have been shown to be time efficient^[16]^. However, the diagnostic test accuracy of different CAD systems is still controversial.

We therefore conducted a systematic review and meta-analysis to:

(1) evaluate the diagnostic accuracy of CAD system based on MRI of the prostate and provides a malignancy assessment;
(2) determine which classifier of CAD system is superior for the diagnosis of PCa;
(3) determine whether the performance of the CAD system depends on the specific regions of the prostate.

## 2 Materials and methods

We conducted our systematic review and meta-analysis according to the Preferred Reporting Items for a Systematic Review and Meta-analysis of Diagnostic Test Accuracy Studies (PRISMA-DTA) guidelines.^[17]^ The study has been registered in PROSPERO, registration number is CRD42019132543. Ethics approval was not required for this systematic review as it involved the collection and analysis of secondary data.

### 2.1 Inclusion and exclusion criteria

Studies were eligible for inclusion if they fulfilled the following criteria:

(1) patients 18 years of age or older and with suspicious clinical symptoms of PCa.
(2) computer-aided system was used to diagnose PCa;
(3) biopsy served as the reference standard;
(4) study data was based on MRI.

Duplicate articles, review articles, editorials, case reports, summaries, animal and cell studies, meta-analyses, letters, editorials, comments, and other irrelevant article types were excluded.

### 2.2 Data sources

Cochrane library, PubMed, EMBASE, and China Biology Medicine disc were systematically searched from their inception to March 2019. We did MeSH and free texts terms searches, as follows: (“prostatic neoplasm∗” OR “prostate neoplasm∗” OR “prostate cancer∗” OR “prostatic cancer∗” OR “prostate tumor∗” OR “prostatic tumor∗”) AND (“artificial intelligence” OR “deep learning” OR “computer-assisted” OR “machine learning” OR “neural network∗” OR “artificial inligence” OR “AI” OR “computational intelligence” OR “machine intelligence” OR “computer reasoning” OR “automated”) AND (“diagnosis” OR “diagnos∗” OR “detection” OR “sensitivity” OR “specificity” OR “accuracy,” “positive likelihood” OR “negative likelihood” OR “ROC”). More search details are shown in Appendix1. In addition, the reference lists of identified studies were manually checked to include other potentially eligible trials. Finally, we transferred all relevant titles and abstracts to Endnote Web for selection.

### 2.3 Study Selection and data extraction

Two authors (X.X.P and Z.X.K) independently screened all titles and abstracts of the retrieved literatures, evaluated potentially relevant full texts, and determined eligibility. Disagreements were resolved through discussion and consensus, or by consulting a third member (L.Y.D) of the review team.

Two reviewers (X.X.P,W.H.P) independently extracted the following information:

1) Basic characteristics of included studies: first author, year of publication, country, patient numbers, patient ages, study design, prostate-specific antigen (ng/mL), testing set, reference standard;
2) The details of different CAD systems: field strength, classifier, Steps of CAD System, Imaging sequence used in system.
3) Diagnostic data: total numbers of true positives, true negatives, false positives and false negatives, as well as accuracy, sensitivity, and specificity of the CAD results.

Discrepancies were discussed and resolved by consensus with a third reviewer.

### 2.4 Methodological quality assessment

Two authors (X.X.P and Z.X.K) independently evaluated the methodological quality of each eligible study using Quality Assessment of Diagnosis Accuracy Study tool, a newly revised quality assessment tool developed specifically for the systematic review of diagnostic accuracy studies,^[18]^ discrepancies were discussed and resolved by consensus, consulting a third reviewer (L.Y.D) if necessary. The tool comprises 4 domains:

(1) patient selection, which describes the method for selecting patients and the patients included;
(2) index test, which describes the test being studied, how it was conducted, and how the results were interpreted;
(3) reference standard, which describes the reference standard test used, how it was conducted, and how the results were interpreted; and
(4) flow and timing, which describe the flow of patient inclusion and exclusion and the interval between the index test and the reference standard.^[19]^

And each question can be answered with “yes,” “no”, or “unclear,” and the level of risk of bias can be judged as “low risk,” “high risk” or “unclear risk” homologous. Finally, Review Manager 5.3 software was used to evaluate the risk of bias of each included study and draw the risk of bias’ figure.

### 2.5 Quality of the evidence

A Grading of Recommendations Assessment, Development and Evaluation (GRADE) approach for diagnostic tests has now been developed, which provides guidance on how to translate accuracy data into a recommendation involving patient-important outcomes.^[20,21]^ We rated the quality of the evidence using the GRADE approach, which considers 5 aspects: risk of bias, indirectness, inconsistency, imprecision, publication bias.

### 2.6 Statistical analyses

We first extracted the 2 × 2 contingency table (true positives, true negatives, false positives and false negatives), some of the primary studies didn’t directly give all the data in the 2 × 2 tables, we calculated the missing data based on the existing data in the text or appendices in each primary study using the calculator in Review Manager 5.3. Using these tables, we determined the true-positive rate (TPR; sensitivity) the true-negative rate (TNR; specificity), forest plots and summary receiver operating characteristic (SROC) curves by Review Manager 5.3. And the stata12.0 software was also used to develop forest plot so as to present the sensitivity and specificity and their pooled results. SROC curves plots each study according to its sensitivity (y-axis) and specificity (x-axis), and each data point represents 1 particular study, and the area under the curve (AUC) was the final comparison indicator. The diagnostic accuracy of CAD was classified according to the AUC as excellent (0.90–1), good (0.80–0.90), fair (0.70–0.80), poor (0.60–0.70), or failed 0.50–0.60.^[22]^

Heterogeneity in test accuracy among studies was explored by using the inconsistency index (*I*^2^ value) and Cochran *Q* statistics for each forest plot, and the difference was considered significant when the P value was less than 0.05. *I*^2^ values greater than 50% indicated substantial heterogeneity in the diagnostic parameters across studies.^[23,24]^

### 2.7 Subgroup-analyses

We conducted subgroup analyses according to the type of classifier of CAD systems used and the different prostate zone (peripheral zone, transitional zone and central gland). Publication biases was investigated by Deek funnel plot.^[25]^

## 3 Results

### 3.1 Literature search

**Figure 1:**
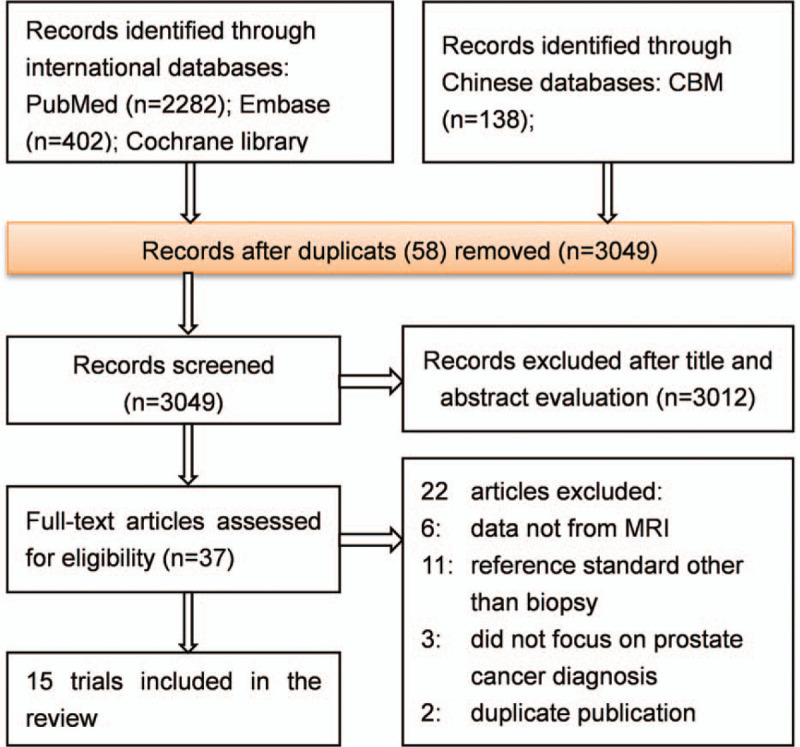
Flow chart shows summary of the literature review process.

Our search retrieved 3107 potentially eligible articles. Of these, 58 were excluded because of duplication, and 3012 on the basis of title or abstract that was irrelevant to the topic, Of the remaining 37 articles, 22 were excluded after reading the full text, and finally 15 studies^[4,11–14,26–35]^ were included in our review. Figure 1 provides an overview of the literature search and study selection.

### 3.2 Characteristics of included studies

The baseline and general characteristics of the included studies are presented in Table 1, the details of CAD system are shown in Table 2. All articles included were retrospective studies. The studies were published between 2007 and 2018. Four studies^[13,28,33,35]^ were performed in the US, 3 in Germany,^[11,14,29]^ 3 in China,^[31,32,34]^ 2 in the Netherlands,^[27,30]^ 1 in France,^[4]^ and 2 in Italy.^[12,26]^ The sample size ranged from 21 to 347. Two studies^[34,35]^ focused on CAD system based on artificial neural networks (ANN), 1 study^[11]^ used a radiomic machine learning classifier, 7^[12,26–28,31–33]^ used support vector machines (SVMs), one^[30]^ used a linear discriminant analysis classifier, and the remaining 4^[4,13,14,29]^ studies did not specify classifiers they used. Only 6 studies^[11,13,28,30,32,35]^ mentioned the training set they used. Nine studies^[11,13,14,28–32,35]^ used 3 Tesla MR scanner to conduct data acquisition, 4 studies^[12,26,33,34]^ used 1.5 Tesla MR scanner, and 2 studies did not reported the type of scanner.

**Table 1:** Characteristics of included studies.

First author	Publication year	Country	Study design	Sample size	Training set	Testing set	Mean Age (yr)	PSA (ng/mL)
Artan^[33]^	2010	USA	retrospective study	21 patients	NR	21 patients	NR	NR
Bonekamp^[10]^	2018	Germany	retrospective study	316 patients	183 patients	133 patients	64.0 ± 7.8	7.5 (5.4–11)
Giannini^[25]^	2015	Italy	retrospective study	56 patients (65 lesions)	NR	56 patients (65 lesions)	64 (60–70)	5.9 (4.9–8.6)
Giannini^[11]^	2016	Italy	retrospective study	58patients	NR	58patients	64 (60–70)	6.7 (5.1–8.4)
Kwak^[12]^	2015	USA	retrospective study	244patients	108 patients	136 patients	63.32 (7.63)	9.71 (10.23)
Litjens^[26]^	2014	Netherlands	retrospective study	347 patients	NR	347 patients	NR	NR
Liu^[27]^	2013	USA	retrospective study	54 patients	36 patients	18 patients	NR	NR
Puech^[28]^	2007	France	retrospective study	100patients	NR	84 patients	NR	>=4
Roethke^[13]^	2016	Germany	retrospective study	45 patients	NR	45 (1102 lesions)	66 (49-77)	7.86 (1.75-39.2)
Thon^[29]^	2017	Germany	retrospective study	79 patients (104 lesions)	NR	79 patients (104 lesions)	64.61 ± 6.64	NR
Vos^[30]^	2012	Netherlands	retrospective study	200 patients	6227candidates	200 patients	60 (50–69)	13.6 (1–58)
Wang^[31]^	2017	China	retrospective study	54 patients	NR	54 patients	74 (65.7–78.3)	23.6 (2.5–56.1)
Yang^[32]^	2017	China	retrospective study	160 patients	1379 negative 300 positive s	160 patients	66.6 ± 9.8	91.99 ± 143.4
Zhao^[34]^	2015	China	retrospective study	71 patients	NR	71 patients	68.8 ± 8.9	11.7 ± 8.1
Zhong^[35]^	2018	USA	retrospective study	140 patients	110patients (169 lesions)	140 patients (216 lesions)	43–80	7.9 ± 12.5

**Table 2:** The details of CAD systems of included studies.

First author	Publication year	Field strength	Classifier	Steps of System
Zhong^[35]^	2018	3T	ANN	input data→ proper pre-processing→predicted probability→ review zone information
Zhao^[34]^	2015	1.5T	ANN	T2-weighted images→ROIs selection→Features extraction→Features selection→ANN classification→ Performance evaluation
Bonekamp^[10]^	2018	3 T	RML	Extract T2W, ADC, B1500 from MRI→3D segmentation→image normalization→feature extraction (first order features, volume/shape features/texture features)→Analysis
Litjens^[26]^	2014	Not specified	SVM	Prostate segmentation→Voxel feature→Vocel calssificatioin→candidate detection→candidate segmentation → candidate feature calculation→candidate classificatior
Puech^[28]^	2007	Not specified	Not specified	Software design→Software design→Semiquantitative analysis
Thon^[29]^	2017	3 T	Not specified	Registration→feature extraction→Feature classifier
Kwak^[12]^	2015	3 T	Not specified	Postprocessing→Feature extraction→Feature selection→ Classification→ Statistical analysis
Roethke^[13]^	2016	3T	Not specified	Feature vector→preparation transformation→predictor→classifier→regularisation→MAI
Giannini^[25]^	2015	1.5 T	SVM	Mp-MR exam→image registration→prostate segmentation→features extraction→features selecction→SVM classifier→Voxel-wise malignancy probability map→candidate selection→FP reduction→candidate segmentation
Yang^[32]^	2017	3 T	SVM	NR
Artan^[33]^	2010	1.5T	SVM	Registration → Segmentation
Wang^[31]^	2017	3T	SVM	NR
Liu^[27]^	2013	3T	SVM	Image Post-processing (T2W2, DWI, DCE, Normalized T2W2, ADC, Ktrans)→registration→patch generation → feature extraction→lesion centroid labelled by radiologist→targeted MRI/TRUS Fusion biopsy→histopathology→SVM classifier training→SVM classifier performance testing
Giannini^[11]^	2016	1.5 T	SVM	MR feature extration→voxel classification (SVM)→Post processing→pre-processing&training construction→texture feature extraction→discretization→feature selection→classifiers training (ANN)→Elements classification→voting system→post processing
Vos^[30]^	2012	3T	LDA	Classification→segmentation→ extracted features

### 3.3 Quality assessment

**Figure 2:**
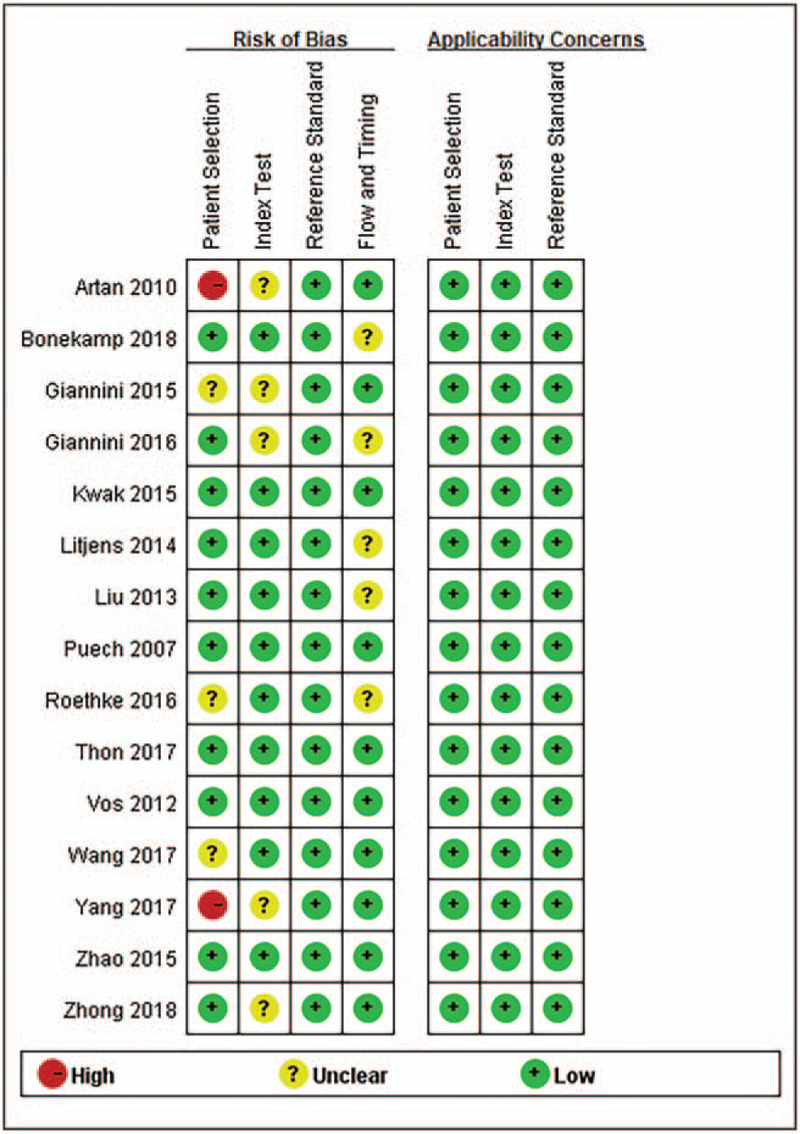
Bar charts showing the result of study quality assessment with QUADAS-2 tool. QUADAS-2 = Quality Assessment of Diagnosis Accuracy Study.

According to the Quality Assessment of Diagnosis Accuracy Study -2 quality assessment results, 7 studies^[11,13,14,27,29,30,34]^ enrolled consecutive samples of patients. All studies avoided inappropriate exclusions. Only 2 studies^[12,26]^ reported the interval between the index test and reference standard. All studies used biopsy as the gold standard. The overall quality of the studies included was qualifying and satisfactory (Fig. 2). The quality of evidence is low, shown in Table 3.

**Table 3:** The quality of evidence (GRADE assessment results).

Sensitivity	0.87 (95% CI: 0.76 to 0.94)								
Sensitivity	0.87 (95% CI: 0.76 to 0.94)								
Outcome			Factors that may decrease certainty of evidence					Effect per 1000 patients tested	
	No of studies (No of patients)	Study design	Risk of bias	Indirectness	Inconsistency	Imprecision	Publication bias	pre-test probability of 0%	Test accuracy
True positives (patients with prostate cancer)	10 studies 846 patients	retrospective study	serious	not serious	serious	not serious	none	0 (0 to 0)	⊕⊕○○ LOW
False negatives (patients incorrectly classified as not having prostate cancer)								0 (0 to 0)	
True negatives (patients without prostate cancer)	10 studies 1787 patients	retrospective study	serious	not serious	serious	not serious	none	760 (620 to 850)	⊕⊕○○ LOW
False positives (patients incorrectly classified as having prostate cancer)								240 (150 to 380)	

### 3.4 Diagnostic accuracy

**Figure 3:**
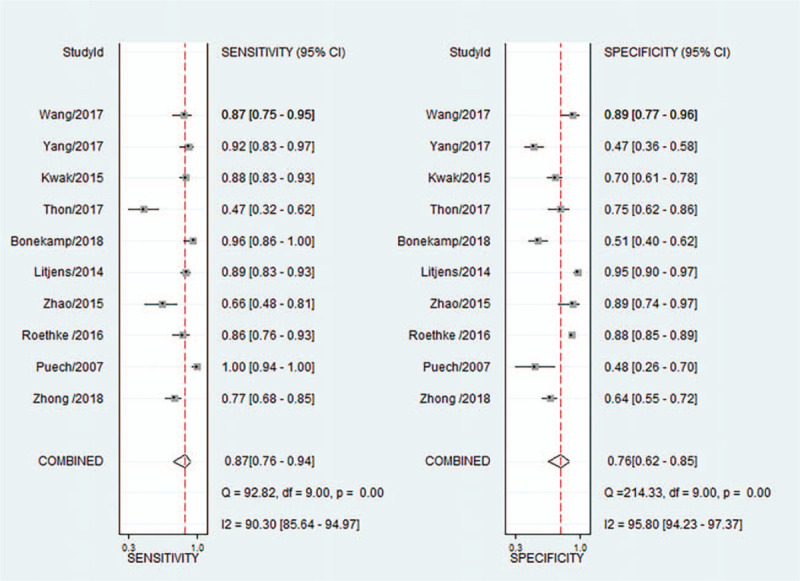
Forest plots show diagnostic performance estimates (sensitivity and specificity) of each study.

**Figure 4:**
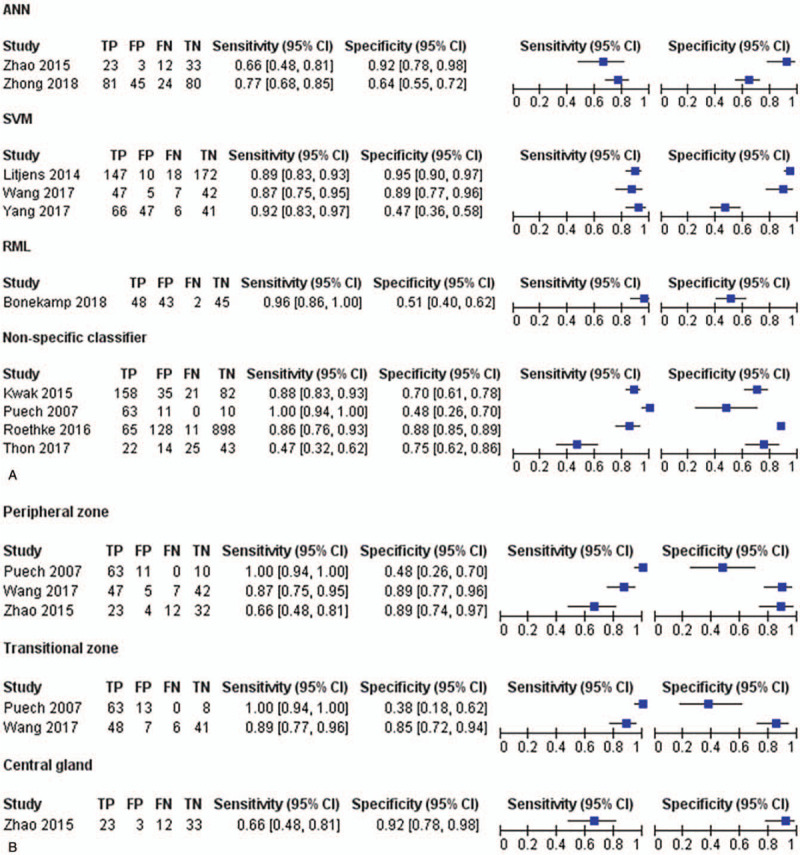
Subgroup analysis of the sensitivity and specificity of CAD system with different classifiers and different prostate zones.

**Figure 5:**
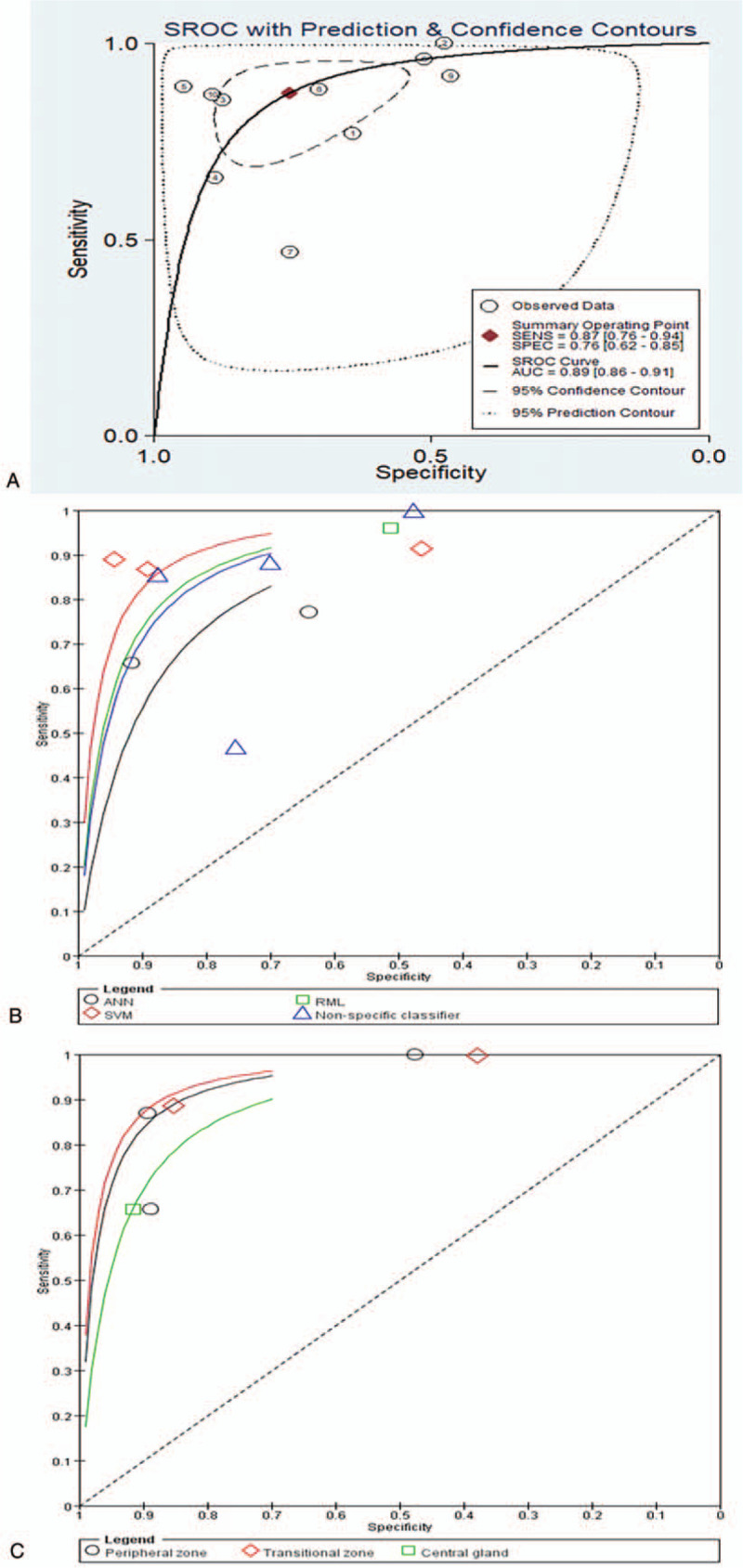
summary receiver operating characteristic (SROC) plots of computer-aided detection for prostate cancer diagnosis based on magnetic resonance imaging with different classifiers and prostate zones.

Ten^[4,11,13,14,27,29,31,32,34,35]^ of the 15 included studies provided specific data to determine the 2x2 tables and were thus eligible for the meta-analysis. The remaining five studies^[12,26,28,30,33]^ all concluded that the CAD method may assist the radiologist to detect PCa locations and could potentially guide to take the biopsy from the most aggressive part of the tumor. Our meta-analysis showed that the sensitivity of CAD system ranged from 0.47 to 1.00 and specificity from 0.47 to 0.89 between the studies. The pooled sensitivity of CAD systems was 0.87 (95% CI, 0.76–0.94) and the pooled specificity was 0.76 (95% CI, 0.62–0.85; Fig. 3). The SROC curve of CAD systems is shown in Figure 5; the AUC was 0.89 (95% CI, 0.86–0.91). Heterogeneity was high in terms of both sensitivity (*I*^2^ = 90.3%) and specificity (*I*^2^ = 95.8%). Subgroup analyses showed that the sensitivity of CAD systems in studies that did not specify classifiers ranged from 0.47 to 1.00, and the specificity from 0.48 to 0.88; the sensitivity of CAD systems based on artificial neural networks classifier ranged from 0.66 to 0.77, and the specificity from 0.64 to 0.92; the sensitivity of CAD using radiomic machine learning classifier was 0.96, and the specificity 0.51; and the sensitivity of CAD using SVM classifier ranged from 0.87 to 0.92, and the specificity from 0.47 to 0.95. In the peripheral zone, the sensitivity ranged from 0.66 to 1.00, and specificity from 0.48 to 0.89; in the transitional zone, sensitivity ranged from 0.89 to 1.00, and specificity from 0.38 to 0.85; and in the central gland, the sensitivity was 0.66, and specificity 0.92. (Fig. 4).

### 3.5 Publication bias

**Figure 6:**
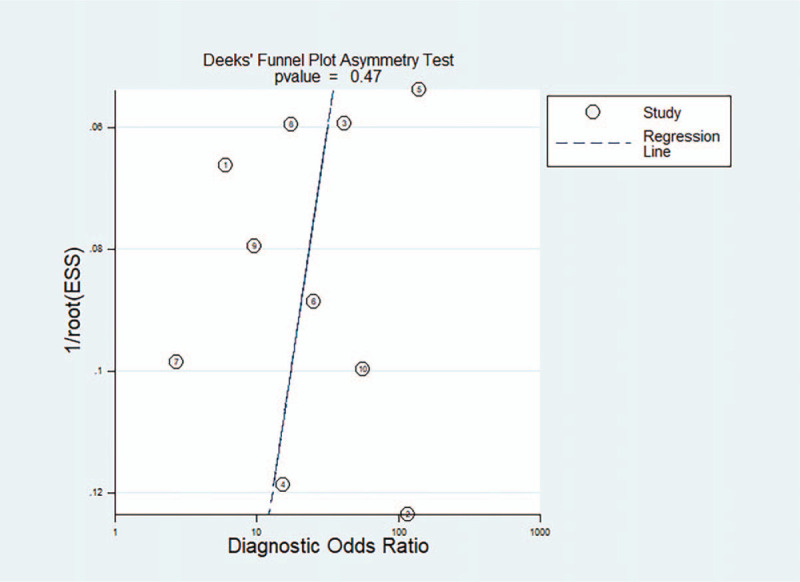
Deeks funnel plot shows asymmetry and presence no publication bias.

The *P*-value for Deek test in this meta-analysis was 0.47, indicating that there was no publication bias. The results of publication bias are shown in Figure 6.

## 4 Discussion

Based on the available evidence, the overall sensitivity of CAD systems for diagnosing PCa ranged from 0.47 to 1.00 and specificity from 0.47 to 0.89, with an AUC of 0.89 (0.86–0.91). Subgroup analyses indicated that the SVM produced the best AUC among the CAD classifiers in the meta-analysis results. The sensitivity and specificity of CAD in the transitional zone was higher than in the peripheral zone and central gland.

A review^[36]^ published in 2014 concluded that kernel-based learning methods such as SVMs showed high sensitivity and specificity and were employed by the majority of research groups for classifying PCa from normal tissue. The conclusion was consistent with our results. But the sample size in both our study and the previous review were small, because many studies did not specify the classifier they used. In our included studies, only 3 studies reported that they used SVM. Therefore, these results are not sufficient to accurately analyze and compare the accuracy of different classifiers. For future studies it is essential to declare the classifiers they use and the overall flow of CAD diagnosis.

The performance of the CAD system may dependent on the location of the tumor in the prostate, for example, central gland, peripheral zone and transitional zone. Among the included studies, only 3 studies^[4,31,34]^ measured the sensitivity and specificity in different prostate zones, other studies did not distinguish the different prostate zones, only a set of data was reported. In our meta-analysis, we conducted subgroup analysis according to different zone of prostate, AUC curve showed that the sensitivity and specificity in the transitional zone was higher than in the peripheral zone and central gland. Therefore, future original studies should consider the influence of different prostate regions on the diagnosis results when exploring the diagnostic accuracy of CAD system for PCa.

A prerequisite of the high accuracy of CAD is the training of the classifier on a database with similar characteristics to the testing database.^[36,37]^ An important limitation of most of the included trials was the lack of description of their classifier training, and it is unclear if this is because the training was not performed, or if it just was not reported. Even if the classifier was trained on scanner data with the same field strength (3T or 1.5T), differences in factors such as technical characteristics, coils, static magnetic field and protocols for the resonance frequency adjustment can lead to differences that could sufficiently affect the outcome. In a thorough review by Wang et al^[36]^ numerous studies with databases varying between 15 and 100 patients were compared, not only in terms of performance but also in terms of the analyzed modalities, field strength, ground truth, the method for candidate lesion generation and applied classifier, revealing a broad heterogeneity. In our meta-analysis, the high heterogeneity among included studies may also be due to the above reasons.

Overall, CAD aided MRI is suitable for screening PCa, because multiple prostate-specific features like peripheral and transition zone distinction, synchronized view of T2-w and T1-wDCEMRI images, analysis of T2-w morphological data or volume assessment were missing in other available software.^[38]^ Additionally, in our meta-analysis, we conducted subgroup analysis according to different zones of the prostate, and the results showed that the sensitivity and specificity of CAD in the transitional zone was different from peripheral zone and central gland. Therefore, it can be explained that different parts of the human body have different sensitivity to screening tools. However, with the exception of CAD - assisted MRI, other screening tools may not recognize it because of poor sensitivity.

### 4.1 Strengths and limitations

Systematic reviews and meta-analyses can provide more reliable evidence than individual trials,^[39,40]^ as their outcomes are derived from all published clinical trials, and as they can be systematically reviewed for the risk of bias.^[41,42]^ We conducted a systemic review of CAD system based on MRI for the diagnosis of PCa using appropriate methodologies and quality assessment tools that may feed into an evidence-based clinical practice. This will be the first systematic review to directly compare the diagnostic accuracy of CAD system based on MRI to a reference standard of PCa.

Because of some limitations, the results of this meta-analysis should be carefully interpreted. First, the number of patients in some studies was relatively small, which can reduce the statistical power. Second, some studies did not report the classifier that the CAD system used, so only few studies could be included in the subgroup analyses comparing the classifiers, which may reduce the reliability of the results.

## 5 Conclusions

Our study indicated that the use of CAD systems to interpret the results of MRI had high sensitivity and specificity in diagnosing PCa. We believe that SVM should be recommended as the best classifier for the CAD system. Whether the performance of the CAD system depends on the specific zones of the prostate needs further investigation.

## Acknowledgments

We would like to thank Janne for helping us to polish and revise grammar spelling.

## Author contributions

**Methodology:** Xinke Zhao, Huiping Wei, Yingdong Li.

**Project administration:** Yingdong Li.

**Resources:** Xiping Xing, Xinke Zhao.

**Software:** Xiping Xing, Huiping Wei.

**Writing – original draft:** Xiping Xing.

**Writing – review & editing:** Yingdong Li.
